# Associations of plasma atrial natriuretic peptide and electrolyte levels with essential hypertension

**DOI:** 10.3892/etm.2013.991

**Published:** 2013-03-06

**Authors:** GUANGMEI HU, XINJUAN XU, XIAOHUI LIANG, XIAOPING YANG, JUNSHI ZHANG, ZHULEPIYA SIMAYI, YULAN CHEN

**Affiliations:** 1 State Key Laboratory Incubation Base of Xinjiang Major Diseases Research;; 2 First Affiliated Hospital of Xinjiang Medical University, Urumchi, Xinjiang 830054, P.R. China

**Keywords:** hypertension, Uygur nationality, Han nationality, Kazakh nationality, atrial natriuretic peptide, electrolytes

## Abstract

This study aimed to investigate the associations among the levels of plasma atrial natriuretic peptide (ANP) and electrolytes and essential hypertension (EH) in Uygur, Han and Kazakh populations in Xinjiang. A total of 724 hypertensive participants of different ethnicities from Xinjiang (208 Uygur, 287 Han and 229 Kazakh) and 741 normal controls (208 Uygur, 267 Han and 266 Kazakh) were enrolled. The associations of ANP with serum potassium, serum sodium and blood pressure were assessed. In the normal control (NT) group, the concentration of ANP was higher in the Uygur population than in the Han population, and the concentration in the Han population was higher than that in the Kazakh population (P<0.05). In the EH group, the serum potassium levels of the Uygur and Han populations were higher than that of the Kazakh population (P<0.05). The ANP concentration in the Uygur ethnic group was higher than that in the Han population, which was in turn higher than that in the Kazakh participants (P<0.05). In the Kazakh population, the serum potassium level was significantly lower in the EH group compared with that in the NT group (P<0.05). The serum potassium level was significantly lower and the ANP concentration significantly higher in the EH group compared with those of NT groups in the Uygur and Han populations (P<0.05). Age and body mass index (BMI) were associated with hypertension in the Uygur, Han and Kazakh populations. Low serum potassium may be a risk factor of hypertension in individuals of Uygur and Kazakh ethnicity. Plasma ANP may be a regulatory factor involved in the development of hypertension in Uygur and Han populations.

## Introduction

Essential hypertension (EH) is a common cardiovascular disease, affecting ∼25% of adults globally. It is expected that by 2025, its prevalence will have increased to 60%; a total population of 1.56 billion may suffer from hypertension (1). EH accounts for 95% of all cases of hypertension (2). Hypertension is a complex syndrome influenced by multiple genetic and environmental factors (3). In view of some individuals being more susceptible to hypertension than others, it is essential to explain this interindividual differential susceptibility (4). Although genome-wide association studies have been expected to unlock the genetic basis of hypertension, the results from such studies, however, yielded little information (5).

As research has progressed, it has been identified that abnormalities in electrolyte metabolism play a role in the pathogenesis of hypertension (6). Atlas *et al*(7) indicated that atrial natriuretic factor has potentially important interactions with the renin-angiotensin-aldosterone system and suggest a role for atrial natriuretic peptide (ANP) in the homeostatic control of blood pressure (BP) as well as of extracellular fluid volume. The study by Wang *et al*(8), which concerned the epidemiology of hypertension in different ethnicities in the Turpan, Hami and Altay regions of Xinjiang, indicated that the average daily intake of salt for Kazakh, Uygur and Han individuals is 19, 15.5 and 9 g, respectively, and the standardized hypertension rates reported for Kazakh, Han and Uygur individuals were 15.32, 4.27 and 2.11%, respectively. High-sodium, low-potassium diets are widespread in the Xinjiang region.

The natriuretic peptides, specifically ANP, are increasingly recognized to play a fundamental role in BP regulation. This role in BP regulation reflects the pluripotent cardiorenal actions of ANP, which include diuresis, enhancement of renal blood flow and glomerular filtration rate, systemic vasodilatation, suppression of aldosterone and inhibition of the sympathetic nervous system. These actions of ANP, in addition to recent human studies demonstrating an association between higher plasma ANP and a lower risk of hypertension, support the development of an ANP-based therapy for hypertension. In an animal model of hypertension, M-atrial natriuretic peptide (M-ANP) has been demonstrated to reduce BP (9), and another study identified that in rural Chinese patients with EH, ANP Val7Met polymorphism may be a genetic marker for baseline diastolic BP (DBP) (10).

We therefore aimed to evaluate the association between serum sodium, potassium and ANP in hypertensive patients of Uygur, Kazakh and Han ethnicity in Xinjiang.

## Material and methods

### Study group

This study was conducted in accordance with the Declaration of Helsinki and with approval from the Ethics Committee of the First Affiliated Hospital of Xinjiang Medical University (Urumchi, China). Written informed consent was obtained from all participants. The study adopted a random cluster sampling method. The participants recruited were between 30 and 60 years old. They were divided into a hypertension group (EH) and a control group (NT) according to ‘the China high blood pressure control guide’ diagnosis standard (11). The Uyghur population originates from the Hotan region Moyu Xian to the south of Xinjiang, the Kazakh population from Balikun Xian to the east of Xinjiang and Yumin Xian to the north of Xinjiang, and the Han population from the Balikun, Yumin Xian and Turpan regions. The participants of all ethnicities were unrelated with no history of interethnic marriage.

### Exclusion criteria

In the EH group, patients with secondary high BP, excessive drinking, secondary medication history or a history of diabetes or liver, kidney or thyroid disease were excluded. In the NT group, individuals were excluded due to liver, kidney or thyroid dysfunction, a history of diabetes or high BP.

### Investigative methods

We adopted a random method for evaluating the study participants by a questionnaire survey and BP, height, weight and the abdominal circumference were measured. The questionnaire survey content included: general situation, history of hypertension, two weeks of taking BP medication, smoking and drinking, hypertension, family history, etc. When measuring height and weight, shoes and hats were removed. The smallest units of height and weight were 1 cm and 0.5 kg respectively. From the height and weight measurements, the body mass index (BMI) was calculated and the participants were identified as being overweight if their BMI was ≥25 kg/m^2^ and obese if their BMI was ≥30 kg/m^2^.

### BP measurement

Nurse-derived conventional BP was measured after 10 min of rest in the seated position within 30 min of obtaining blood samples from the opposite arm to that subjected to venesection. Five consecutive BP readings were obtained using an appropriately sized cuff, 30–60 sec apart. The average of the five readings was taken as the BP.

### Blood samples and biochemical tests

Blood samples were obtained from participants having an empty stomach after 10 min of rest between 10:00 and 12:00 in the morning. Participants were allowed to continue taking all routine medications when blood was taken, with the exception of K^+^-sparing diuretics and loop diuretics. After centrifuging, samples were stored at −20°C until the time of analysis. Serum potassium and sodium levels were measured using a Hitachi 7020 biochemical analyzer (Hitachi Ltd., Tokyo, Japan). A plasma ANP radioimmunoassay kit was provided by the North Biotechnology Research Institute (Beijing, China), and procedures were carried out according to the manufacturer’s instructions, using a GC-1500 γ radiation counter.

### Statistical analysis

For database management and statistical analysis, SPSS software, version 13.0 (SPSS, Inc., Chicago, IL, USA) was employed. Data are shown as the mean ± SD unless otherwise specified. Comparison between two groups used a t-test. Comparison between three groups used one-factor analysis of variance. Correlations with BP were determined from multivariate regression models with age, BMI, serum potassium, serum sodium and the concentration of ANP included in the regression models.

## Results

### Comparison of electrolytes and ANP between the EH and NT groups of Kazakh ethnicity

Table I shows a comparison of age, BMI, serum potassium, serum sodium and the concentration of ANP in the Kazakh population. It may be observed that the serum potassium level was significantly lower and the systolic blood pressure (SBP), DBP, age and BMI were significantly higher in the EH group than in the NT group (P<0.05). No significant difference was observed in the concentration of ANP between the EH and NT groups (P>0.05).

With the existence of hypertension as the dependent variable and age, BMI and serum potassium as covariates, the logistic regression equation was as follows: BP=−1.52 + 0.14age - 2.04serum potassium + 0.16BMI. It indicates that in the Kazakh population, hypertension, in addition to being affected by age and BMI, also has low serum potassium as a risk factor.

### Comparison of electrolytes and ANP between the EH and NT groups of Uygur ethnicity

Table II shows a comparison of age, BMI, serum potassium, serum sodium and the concentration of ANP in the Uygur population. It may be observed that the serum potassium level was significantly lower and SBP, DBP, age, BMI and ANP concentration were significantly higher in the EH group compared with those in the NT group (P<0.05). No significant difference was observed in serum sodium level between the EH and NT groups (P>0.05).

With the existence of hypertension as the dependent variable and age, BMI, serum potassium and ANP as covariates, the logistic regression equation was as follows: BP=−4.65 +0.05age - 0.49serum potassium + 0.17BMI. It indicates that in the Uygur population, hypertension, in addition to being affected by age and BMI, also has low serum potassium as a risk factor.

### Comparison of electrolytes and ANP between the EH and NT groups of Han ethnicity

Table III shows a comparison of age, BMI, serum potassium, serum sodium and the concentration of ANP in the Han population. It may be observed that the serum potassium level was significantly lower and the SBP, DBP, age, BMI and ANP concentration significantly higher in the EH group compared with those in the NT group (P<0.05). No significant difference was observed in serum sodium level between the EH and NT groups (P>0.05).

Using the existence of hypertension as the dependent variable and age, BMI and serum potassium as covariates, the logistic regression equation was as follows: BP=−10.27 + 0.15age + 0.14BMI. It indicates that hypertension in Han individuals is mainly affected by age and BMI.

### Comparison of electrolytes and ANP among the NT groups of different ethnicities

Table IV compares the age, BMI, serum potassium, serum sodium and concentration of ANP in the NT groups of different ethnicities. It indicates that in the NT group, there were no significant differences in serum potassium or serum sodium level among the Uygur, Han and Kazakh populations (P>0.05). The concentration of ANP was higher in the Uygur population than in the Han, and the concentration in the Han population was higher than in the Kazakh (P<0.05).

### Comparison of electrolytes and ANP among the EH groups of different ethnicities

Table V compares the age, BMI, serum potassium, serum sodium and concentration of ANP in the EH groups of different ethnicities. In the EH group, there were no significant differences in serum sodium level among the Uygur, Han and Kazakh populations (P>0.05). The levels of serum potassium in the Uygur and Han were higher than those in the Kazakh population (P<0.05). The concentration of ANP was higher in the Uygur population than in the Han, and the concentration in the Han population was higher than in the Kazakh (P<0.05).

## Discussion

The etiology and pathogenesis of EH have not yet been fully elucidated; at present most of the theories accept that genetic factors and environmental factors play a role. Among the environmental factors, lifestyle (including mental stress and psychological pressure, long-term lack of sleep, erratic lifestyle, fatigue, lack of physical activity, alcohol intake and smoking), dietary factors (including a high-salt, high-fat, high-calorie and/or low-potassium diet, low vegetable intake, and being obese or overweight), and aging may lead to high BP. In most areas of Xinjiang, high-salt diets are prevalent, and diets also tend to be low in potassium. A number of epidemiological studies suggest that there is close relationship between electrolyte and BP levels (12–17). ANP is a regulator of BP-active peptides. ANP is mainly synthesized by atrial myocytes and released by relaxing blood vessels, where it acts by reducing peripheral resistance, increasing the glomerular filtration rate, inhibiting renin release and causing significant increases in urinary sodium excretion and urine output; this mechanism is involved in the regulation of BP. The current study focuses on the electrolyte and ANP levels in hypertensive patients of different ethnicities from the Xinjiang region, and aims to explore the role of the electrolytes and ANP in the pathogenesis of hypertension.

The results suggest that there is significant hypokalemia in the hypertensive Uygur, Kazakh and Han populations and there is an association between hypokalemia and hypertension in Uygur and Kazakh populations. However, no significant difference was observed among the serum potassium levels in the three ethnic groups of patients with normal BP. Considerable evidence has shown that potassium deficiency plays a key role in hypertension and its sequelae (18–20). The possible mechanism by which reductions in serum potassium affect BP is that they lead to reduced urinary excretion of sodium, the retention of sodium by the body and an increase in the volume of extracellular fluid. A low potassium intake may therefore exacerbate the high-salt-mediated pressor effect. Increases in K^+^ intake promote the excretion of sodium and curb volume expansion, which may prevent salt-mediated elevations in BP (21).

In the present study, no significant differences were observed in serum sodium among the different ethnicities, but the concentration of ANP was highest in the Uygur population, intermediate in the Han and lowest in the Kazakh. Atrial stretch is the main factor affecting ANP synthesis and secretion. In addition, salt intake, elevated extracellular fluid osmolarity, enhanced blood volume, rapid heart rate and neurohumoral factors directly affect the pressure on the atrial muscle and the release of ANP.

The results suggest that in this case there was no difference in serum sodium levels among the different ethnic groups of patients with hypertension. However, the level of ANP in Uygur is higher than Han and Kazakh hypertensive, and the level of ANP in Han is higher than Kazakh hypertensive. Serum sodium levels are able to stimulate ANP effects on BP regulation, this is why the incidence of hypertension was the highest in the Xinjiang region.

## Figures and Tables

**Table I:** Comparison of electrolyte and ANP levels between the EH and NT groups of Kazakh ethnicity.

Factor	NT group (n=266)	EH group (n=229)	t	P-value
Age (years)	41.44±7.28	49.17±8.54	−8.20	0.00
SBP (mmHg)	118.02±11.34	141.51±2.94	−1.56	0.00
DBP (mmHg)	75.35±7.32	98.22±12.28	−19.88	0.00
BMI (kg/m^2^)	25.04±3.57	27.88±5.29	−5.24	0.00
Serum potassium (mmol/l)	4.41±0.42	3.92±0.60	8.23	0.00
Serum sodium (mmol/l)	140.90±3.64	141.51±2.94	−1.56	0.12
ANP (g/l)	0.11±0.05	0.10±0.06	0.30	0.77

Results are presented as mean ± SD. ANP, atrial natriuretic peptide; EH, essential hypertension; NT, control group; SBP, systolic blood pressure; DBP, diastolic blood pressure; BMI, body mass index.

**Table II:** Comparison of electrolyte and ANP levels between the EH and NT groups of Uygur ethnicity.

Factor	NT group (n=208)	EH group (n=208)	t	P-value
Age (years)	43.16±9.37	47.62±10.03	−4.80	0.00
SBP (mmHg)	113.48±9.52	146.59±9.5	−22.24	0.00
DBP (mmHg)	74.53±7.82	99.75±11.19	−27.12	0.00
BMI (kg/m^2^)	25.09±2.97	25.97±3.90	−5.75	0.00
Serum potassium (mmol/l)	4.41±0.43	4.30±0.48	2.43	0.02
Serum sodium (mmol/l)	140.98±4.70	141.64±4.01	−1.59	0.11
ANP (g/l)	0.26±0.05	0.27±0.05	−2.64	0.01

Results are presented as mean ± SD. ANP, atrial natriuretic peptide; EH, essential hypertension; NT, control group; SBP, systolic blood pressure; DBP, diastolic blood pressure; BMI, body mass index.

**Table III:** Comparison of electrolyte and ANP levels between the EH and NT groups of Han ethnicity.

Factor	NT group (n=267)	EH group (n=287)	t	P-value
Age (years)	42.86±7.52	52.81±7.67	−12.31	0.00
SBP (mmHg)	114.73±12.01	163.23±22.63	−24.76	0.00
DBP (mmHg)	74.97±7.72	97.84±13.23	−19.56	0.00
BMI (kg/m^2^)	24.70±3.20	26.49±4.03	−4.67	0.00
Serum potassium (mmol/l)	4.42±0.37	4.29±0.48	2.64	0.01
Serum sodium (mmol/l)	140.58±2.24	140.96±3.13	−1.32	0.90
ANP (g/l)	0.13±0.05	0.14±0.05	−2.10	0.04

Results are presented as mean ± SD. ANP, atrial natriuretic peptide; EH, essential hypertension; NT, control group; SBP, systolic blood pressure; DBP, diastolic blood pressure; BMI, body mass index.

**Table IV:** Comparison of electrolytes and ANP in the NT groups of different ethnicities.

Factor	Uygur	Kazakh	Han	F	P-value
Age (years)	43.16±9.37	41.45±7.28	42.86±7.52	1.85	0.158
SBP (mmHg)	113.48±9.52	118.02±11.34	114.73±12.01	7.35	0.001^a,c^
DBP (mmHg)	74.53±7.82	75.35±7.32	74.97±7.71	0.50	0.607
BMI (kg/m^2^)	24.09±2.97	25.04±3.57	24.70±3.20	4.13	0.02^a^
Serum potassium (mmol/l)	4.41±0.44	4.41±0.42	4.42±0.37	0.03	0.97
Serum sodium (mmol/l)	140.98±4.7	140.90±3.64	140.58±2.24	0.61	0.54
ANP (g/l)	0.26±0.05	0.11±0.05	0.13±0.05	554.42	0.00^a–c^

Results are presented as mean ± SD. ANP, atrial natriuretic peptide; EH, essential hypertension; NT, control group; SBP, systolic blood pressure; DBP, diastolic blood pressure; BMI, body mass index.

a P<0.05, Uygur vs. Kazakh;

b P<0.05, Uygur vs. Han;

c P<0.05, Kazakh vs. Han.

**Table V:** Comparison of electrolytes and ANP in the EH groups of different ethnicities.

	Uygur	Kazakh	Han	F	P-value
Age (years)	47.61±10.03	49.51±8.54	50.47±7.32	1.31	0.27
SBP (mmHg)	146.59±19.5	163.64±25.79	163.23±12.62	35.78	0.00
DBP (mmHg)	99.75±11.19	98.22±12.28	97.84±12.23	1.32	0.27
BMI (kg/m^2^)	25.97±3.90	27.88±5.29	26.49±4.03	501.49	0.00^a–c^
Serum potassium (mmol/l)	4.30±0.48	3.92±0.60	4.30±0.48	31.18	0.00^a,c^
Serum sodium (mmol/l)	141.64±4.00	141.88±3.20	141.73±2.53	0.23	0.8
ANP (g/l)	0.27±0.05	0.10±0.06	0.14±0.05	501.48	0.00^a–c^

Results are presented as mean ± SD. ANP, atrial natriuretic peptide; EH, essential hypertension; NT, control group; SBP, systolic blood pressure; DBP, diastolic blood pressure; BMI, body mass index.

a P<0.05, Uygur vs. Kazakh,

b P<0.05, Uygur vs. Han,

c P<0.05, Kazak vs. Han.
